# *Tespa1* Deficiency Dampens Thymus-Dependent B-Cell Activation and Attenuates Collagen-Induced Arthritis in Mice

**DOI:** 10.3389/fimmu.2018.00965

**Published:** 2018-05-14

**Authors:** Yunliang Yao, Wei Huang, Xiaoyu Li, Xiawei Li, Jin Qian, Hui Han, Hui Sun, Xiangli An, Linrong Lu, Hongxing Zhao

**Affiliations:** ^1^Program in Molecular and Translational Medicine (PMTM), School of Medicine, Huzhou University, Huzhou, China; ^2^The Children’s Hospital, Zhejiang University School of Medicine, Hangzhou, China; ^3^The Second Affiliated Hospital, Zhejiang University School of Medicine, Hangzhou, China; ^4^First Affiliated Hospital, Huzhou University, Huzhou, China; ^5^School of Medicine, Institute of Immunology, Zhejiang University, Hangzhou, China

**Keywords:** tespa1, PLCG2, B-cell, collagen-induced arthritis, CD40

## Abstract

Thymocyte-expressed, positive selection-associated 1 (*Tespa1*) plays an important role in both T cell receptor (TCR)-driven thymocyte development and in the FcεRI-mediated activation of mast cells. Herein, we show that lack of *Tespa1* does not impair B cell development but dampens the *in vitro* activation and proliferation of B cells induced by T cell-dependent (TD) antigens, significantly reduces serum antibody concentrations *in vivo*, and impairs germinal center formation in both aged and TD antigen-immunized mice. We also provide evidence that dysregulated signaling in *Tespa1*-deficient B cells may be linked to CD40-induced TRAF6 degradation, and subsequent effects on 1-phosphatidylinositol-4,5-bisphosphate phosphodiesterase gamma-2 (PLCγ2) phosphorylation, MAPK activation, and calcium influx. Furthermore, we demonstrate that *Tespa1* plays a critical role in pathogenic B cells, since *Tespa1*-deficient chimeric mice showed a lower incidence and clinical disease severity of collagen-induced arthritis. Overall, our study demonstrates that *Tespa1* is essential for TD B cell responses, and suggests an important role for *Tespa1* during the development of autoimmune arthritis.

## Introduction

B lymphocytes play crucial roles in adaptive immunity by recognizing foreign antigens and eliciting appropriate host protective responses. The developmental fate of B cells, as well as their function during immune responses, is critically regulated by the B-cell receptor (BCR). BCR signaling is initiated by antigen ligation, which triggers the formation of the BCR signalosome, a multifunctional protein complex that includes the protein tyrosine kinase Lyn, spleen tyrosine kinase (Syk), B cell linker protein (BLNK), Bruton agammaglobulinemia tyrosine kinase (Btk), phospholipase Cγ2 (PLCγ2), and inositol 1,4,5-trisphosphate receptor type 2 (IP3R2), ultimately leading to calcium mobilization and the activation of several downstream pathways (1–5). In addition, during thymus-dependent B-cell activation, several coreceptors such as CD40, CD19, CD22, CD21, and FcγRIIB, act to quantitatively modify BCR signaling. The signals generated by the costimulatory receptors are the means through which T cell help modulates BCR signaling (6–8). CD40 is one of the most important coreceptors, and plays a crucial role during T cell-dependent (TD) B cell activation, immunoglobulin class switching, and the development of humoral memory. The signaling pathways emanating from the BCR and CD40 can cooperate in a synergistic or additive manner, but the molecular mechanisms underlying these interactions are not completely understood. CD40 is a member of the TNFR family, and several members of the TNFR-associated factor (TRAF) family have been shown to bind to CD40 and serve as adaptor proteins in the CD40 signaling pathway. TRAFs clearly have important roles in B cell regulation, but the nature of these roles is still unresolved (9–14).

Thymocyte-expressed, positive selection-associated 1 (*Tespa1*) was originally identified as a critical signaling molecule involved in T cell selection and maturation. *Tespa1* regulates T cell receptor (TCR) signaling through direct binding to PLCγ1 and IP3R1, thereby facilitating TCR-induced calcium signals and thymocyte development. It also participates in mitochondrial Ca^2+^ uptake *via* mitochondria-associated ER membranes in T cells (15–18). Furthermore, *Tespa1* was also found to negatively regulate FcεRI-mediated signaling and mast cell-mediated allergic responses (18). Besides T cells and mast cells, *Tespa1* is also highly expressed in most subsets of peripheral B cells, suggesting a potential role in B cell function (19, 20).

In this study, we show that the absence of *Tespa1* does not impair B cell development, but significantly reduces the activation and proliferation of B cells induced by TD antigens, both *in vitro* and in bone marrow chimeras with *Tespa1*-deficient B cells (*Tespa1*^B−/−^). We also found a potential role for *Tespa1* in the stabilization of TRAF 6 and the phosphorylation of PLCγ2 induced by CD40.

Finally, since B cells or some B cell subpopulations play crucial roles in the development of rheumatoid arthritis (RA) in humans and of collagen-induced arthritis (CIA) in mice (21–25), we employed CIA as a model to evaluate the role of *Tespa1* in B cell-associated autoimmune diseases, and found that *Tespa1*-deficient chimeras showed a lower incidence and clinical disease severity index of autoimmune arthritis. This suggests that *Tespa1* is a potential therapeutic target in human RA.

## Materials and Methods

### Ethics Statement

This investigation was conducted in accordance with the ethical standards of the Declaration of Helsinki, followed national and international guidelines and was approved by the review board of the School of Medicine, Huzhou University.

### Animals and Immunization

*Tespa1*^−/−^ mice were generated by homologous recombination-mediated gene targeting at the Shanghai Research Center for Model Organisms, as previously described in Ref. (16). Mice on a mixed 129 × C57BL/6 background were backcrossed onto the C57BL/6 background for 6–8 generations. B6.129S2-Igh-6tm1Cgn (μMT) mice were a kind gift from Qi Hai (Tsinghua University, Beijing, China). For the experiments with mixed bone marrow chimeras, C57BL/6 mice were lethally irradiated (8.5 Gy) and reconstituted with a mixed suspension of μMT (80%) and *Tespa1*^+/+^ or *Tespa1*^−/−^ (20%) bone marrow cells at least 8 weeks before immunization. All mice were housed at the Zhejiang University Laboratory Animal Center. Animal experiment protocols were approved by the Review Committee of Zhejiang University School of Medicine and were in compliance with institutional guidelines. For TD responses, mice were immunized with 100 µg of NP-KLH (Biosearch Technologies, Novato, CA, USA) mixed with Imject alum (Thermo Fisher Scientific, MA, USA) on days 0 (primary immunization) and 21 (secondary immunization). For TI responses, 20 ng of NP-LPS or 20 µg of NP-Ficoll (Biosearch Technologies, Novato, CA, USA) were injected intraperitoneally.

### Antibodies

The following antibodies were used in flow cytometry experiments: FITC-conjugated antibodies to mouse CD5 (53-7.3), IgD (11-26c.2a), CD21/35 (4E3), CD44 (IM7), CD40 (HM40-3) and CD69 (H1.2F3); PE-conjugated antibodies to mouse IgM (RMM-1), CD27 (lg.3a10), CD25 (PC61), CD80 (16-10A1), MHC class II (I-A/I-E) (M5/114.15.2) and CD45.2 (104); PE/Cy5-conjugated antibodies to mouse CD45R/B220 (RA3-6B2) and CD19 (6D5); and APC-conjugated antibodies to mouse CD138 (281-2), CD86 (GL-1), CD45.1 (A20), and CD21/35 (4E3). All antibodies were purchased from BioLegend (San Diego, CA, USA), except the FITC-conjugated antibodies to mouse CD21/35 (4E3) and CD93 (AA4.4), and the APC-conjugated antibody to mouse CD23 (B3B4), which were from Ebioscience (San Diego, CA, USA); as well as the FITC-conjugated antibody to mouse GL-7 (GL7) and the PE-conjugated antibody to mouse CD95/Fas (Jo2), which were from BD Biosciences (San Diego, CA, USA). For immunoblots, anti-mouse Lyn, anti-mouse phospho-Syk (Try532), TRAF2/3/6 antibody were purchased from Santa Cruz Biotechnology (Santa Cruz, CA, USA), anti-mouse Akt, anti-mouse phospho-Akt (Ser473), anti-mouse IκBa, anti-mouse PLCγ2, anti-mouse phospho-PLCγ2 (Tyr759), anti-mouse JNK2, anti-mouse phospho-JNK (Thr183/Tyr185), anti-mouse p44/42 MAPK (Erk1/2), anti-mouse phospho- p44/42 MAPK (Erk1/2) (Thr202/Tyr204), anti-mouse p38 MAPK, and anti-mouse phospho-p38 MAPK (Thr180/Tyr182) antibodies were purchased from Cell Signaling Technology (Danvers, MA, USA). The anti-mouse GRB2, anti-mouse phospho-IκBa (Ser32), anti-mouse BLNK, anti-mouse Btk, anti-mouse phospho-GRB2 (Tyr614), anti-mouse phospho-BLNK (Tyr84), and anti-mouse phospho-Btk (Tyr223) antibodies were from Beijing 4A Biotechnology (Beijing, China). The anti-mouse Syk and anti-mouse NLAT (LAB) antibodies were from BioLegend (San Diego, CA, USA). The anti-mouse phospho-Lyn (Tyr507) antibody was from Abcam (Cambridge, MA, USA), the anti-mouse phospho-NLAT (LAB) (Tyr136) antibody was from Thermo Fisher Scientific (Waltham, MA, USA), and the anti-mouse GAPDH monoclonal antibody was from CWbiotech (Beijing, China).

### Flow Cytometry Analysis

Single-cell suspensions were prepared from bone marrow isolated from the tibia and femur of one leg, and from the spleen and peritoneal cavity using standard procedures. Following red blood cell lysis, Fc receptors were blocked with anti-CD16/32 Ab (2.4G2), and cells were stained with the antibodies mentioned above in 0.5% FBS in PBS. For the proliferation assays, lymphocytes were loaded with carboxyfluorescein succinimidyl ester (CFSE) (Invitrogen, Carlsbad, CA, USA), stimulated as described, and cell divisions were assessed after 3 days by Flow cytometry. Data were collected on a FACSCanto™ II (BD Biosciences San Jose, CA, USA) instrument and analyzed using FlowJo software (Tree Star, Ashland, OR, USA).

### Cell Enrichment and Stimulations

Splenocytes were harvested from *Tespa1*^+/+^ and *Tespa1*^−/−^ mice and B cells were enriched by negative selection using the mouse B Lymphocyte Enrichment Set-DM from BD Biosciences (San Diego, CA, USA), following the manufacturer’s guidelines. Enriched B cells were cultured in DMEM supplemented with 100 mM nonessential amino acids, 20 mM HEPES buffer, 10% FCS, 100 U/ml penicillin, 100 mg/ml streptomycin, 2 mM l-glutamine, and 100 mM 2-ME. Unless indicated otherwise, cells were stimulated with 10 µg/ml of anti-IgM F(ab′)_2_ (Jackson Immunoresearch, West Grove, PA, USA), 10 µg/ml of LPS (Sigma-Aldrich, St. Louis, MO, USA), or 10 µg/ml of anti-mouse CD40 antibody (BioLegends, San Diego, CA, USA).

### Ca^2+^ Flux

Splenocyte suspensions at a density of 5 × 10^6^ cells/ml were loaded with 4 mg/ml of Fluo4 (Invitrogen, Carlsbad, CA, USA) in RPMI without sera for 1 h at 37°C and washed twice with calcium-free Hank’s buffered saline (HBSS; pH 7.4). Cells were labeled with PE/Cy5-conjugated anti-B220 for identification of B cells. After washing, cells were suspended at 2 × 10^6^ cells/ml in calcium-free HBSS, and prewarmed at 37°C for 10 min. Next, 10 µg/ml of anti-mouse CD40 antibody was added to induce calcium flux during the first 60 s, followed by CaCl_2_ at a final concentration of 2 mM for the next 180 s and 1 µM ionomycin (Sigma-Aldrich, St. Louis, MO, USA) for an additional 60 s. Mean fluorescence ratios were plotted after analysis with FlowJo software (Tree Star, Ashland, OR, USA).

### ELISA

Total serum Ig levels were quantified by ELISA using the Mouse Ig Isotyping ELISA Ready-Set-Go! kit (ebioscience, San Diego, CA, USA), according to the manufacturer’s instructions. The concentrations were calculated based on the OD450 values obtained with serial dilutions, using values in the linear portion of the response curve to calculate the serum Ab levels. NP-specific and CII-specific ELISAs were performed following similar methods, but plates were coated with 10 μg/ml of NP-BSA (Biosearch Technologies, Novato, CA, USA) or 5 µg/ml of bovine collagen type II (Sigma-Aldrich, St. Louis, MO, USA).

### Immunohistochemistry and Histological Analysis

Germinal centers (GCs) were stained in splenic paraffin sections using biotinylated peanut agglutinin (PNA), streptavidin-HRP, and the diaminobenzidine method. For histopathologic analysis of joint tissues, whole ankles, and ankle joints were fixed in 10% formalin for 3 days. After decalcification for 18 days in Cal-Ex II (Fisher Scientific, Springfield, NJ, USA), the specimens were processed for paraffin embedding. Tissue sections (5 µm) were stained with H&E for microscopic evaluation.

### Immunoprecipitation and Immunoblot Analysis

Cells stimulated as described were lysed with NP-40 lysis buffer (Beyotime Biotechnology; Shanghai, China), containing 50 mM Tris (pH 7.4), 150 mM NaCl, 1% NP-40, 10 mM PMSF, protease inhibitor cocktail, and phosphatase inhibitor cocktail (Cwbiotech, Beijing, China). The lysates were incubated on ice for 20–30 min, vortexed extensively, and centrifuged (13,000 rpm × 10 min, 4°C). Samples were then boiled for 5 min, subjected to 10% SDS-PAGE, blotted, probed with commercially available antibodies, and bound antibodies detected using the Tanon 5500 enhanced chemiluminescence detection system (Tanon, Shanghai, China).

### Induction of CIA

For CIA induction, *Tespa1*^B+/+^ and *Tespa1*^B−/−^ chimeras were injected subcutaneously at the base of the tail with 100 µg of bovine collagen type II (Sigma-Aldrich, St. Louis, MO, USA) emulsified in Freund’s complete adjuvant (Chondrex, Seattle, WA, USA), followed 21 days later by a booster injection of the same bovine collagen type II (100 µg) emulsified in Freund’s incomplete adjuvant (Sigma-Aldrich, St. Louis, MO, USA), *via* the same route and following the protocol described by Inglis et al. (26). To assess the severity of arthritis, clinical symptoms were evaluated by means of a five-point scale: grade 0 = no swelling; grade 1 = paw with detectable swelling in a single digit; grade 2 = paw with swelling in more than one digit; grade 3 = paw with swelling of all digits and instep; and grade 4 = severe swelling of the paw and ankle.

### Statistics

Differences between groups were analyzed by means of Student’s *t* test. A *p* value <0.05 was considered significant, *p < 0.05, **p < 0.01.

## Results

### Normal B Cell Development in the Absence of *Tespa1*

**Figure 1:**
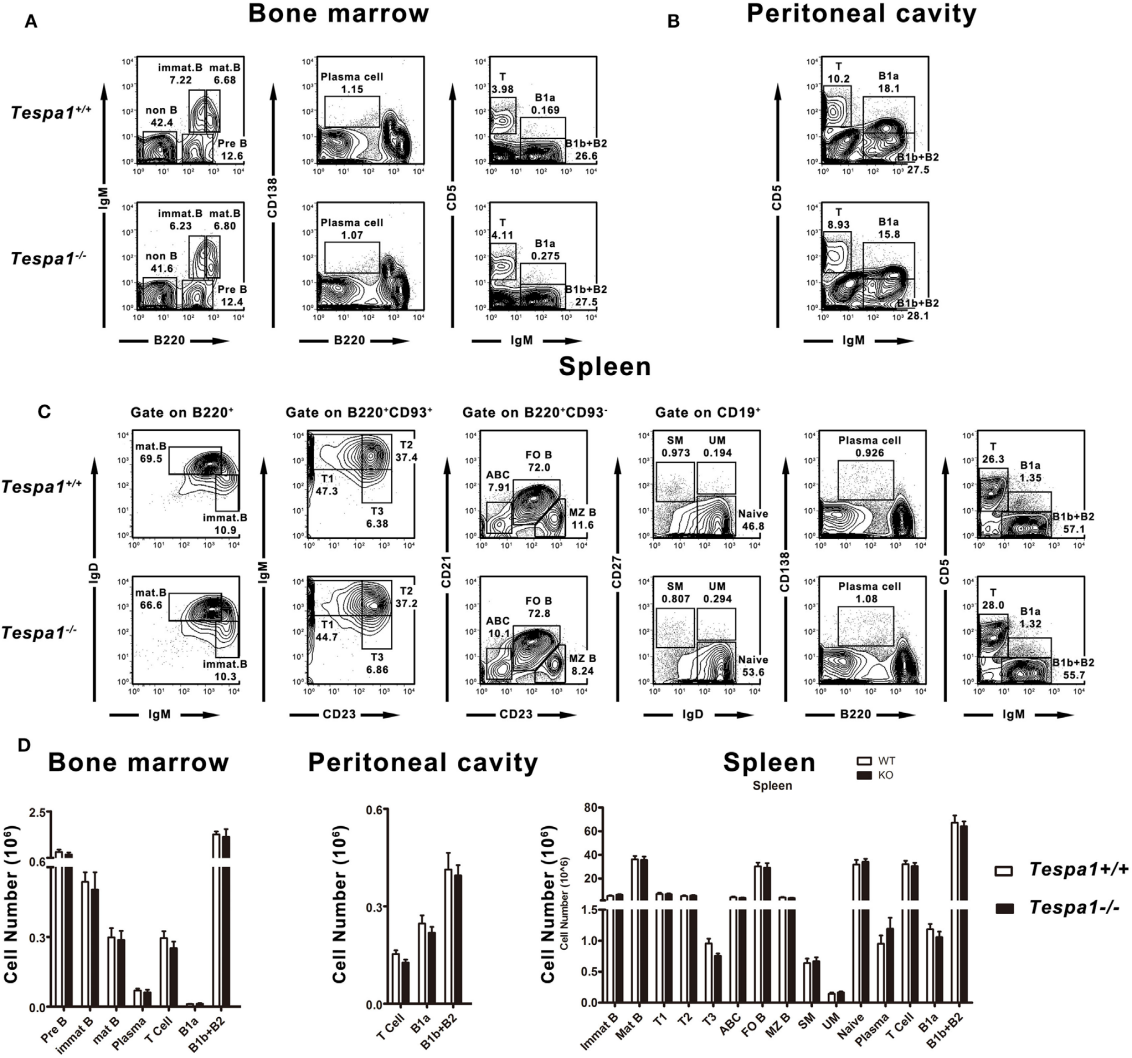
Normal B cell development in *Tespa1*-deficient mice. FACS profiles of B cell subsets present in **(A)** bone marrow, **(B)** peritoneal cavity, and **(C)** spleen of *Tespa1*-deficient and wild-type control mice. **(D)** Numbers and percentages of B cell subsets in the indicated organs of *Tespa1*-deficient and WT mice. Data represent five mice per group. Similar results were obtained in three independent experiments.

To investigate whether *Tespa1* is required for B cell development, we used flow cytometric analysis to quantify the number of developing and mature B cells in lymphoid tissues of *Tespa1*-deficient and wild-type (WT) mice. We found that the numbers of pro-B, pre-B, immature B, mature B, B1, and plasma cells were unaltered in the bone marrow of these mice (Figures 1A,D). Furthermore, there was also no change in the number of B1 cells in the peritoneal cavity (Figures 1B,D). In addition, we examined various B cell subsets in the spleen, and found that the lack of *Tespa1* did not alter the numbers of mature B cells, immature B cells, T1, T2, T3 B cells, age-associated B cells (24), follicular B cells, marginal zone B cells, switched memory B cells, unswitched memory B cells, plasma cells, or B1 cells (Figures 1C,D).

### Aged *Tespa1*-Deficient Mice Have Lower Baseline Concentrations of Serum Immunoglobulins

**Figure 2:**
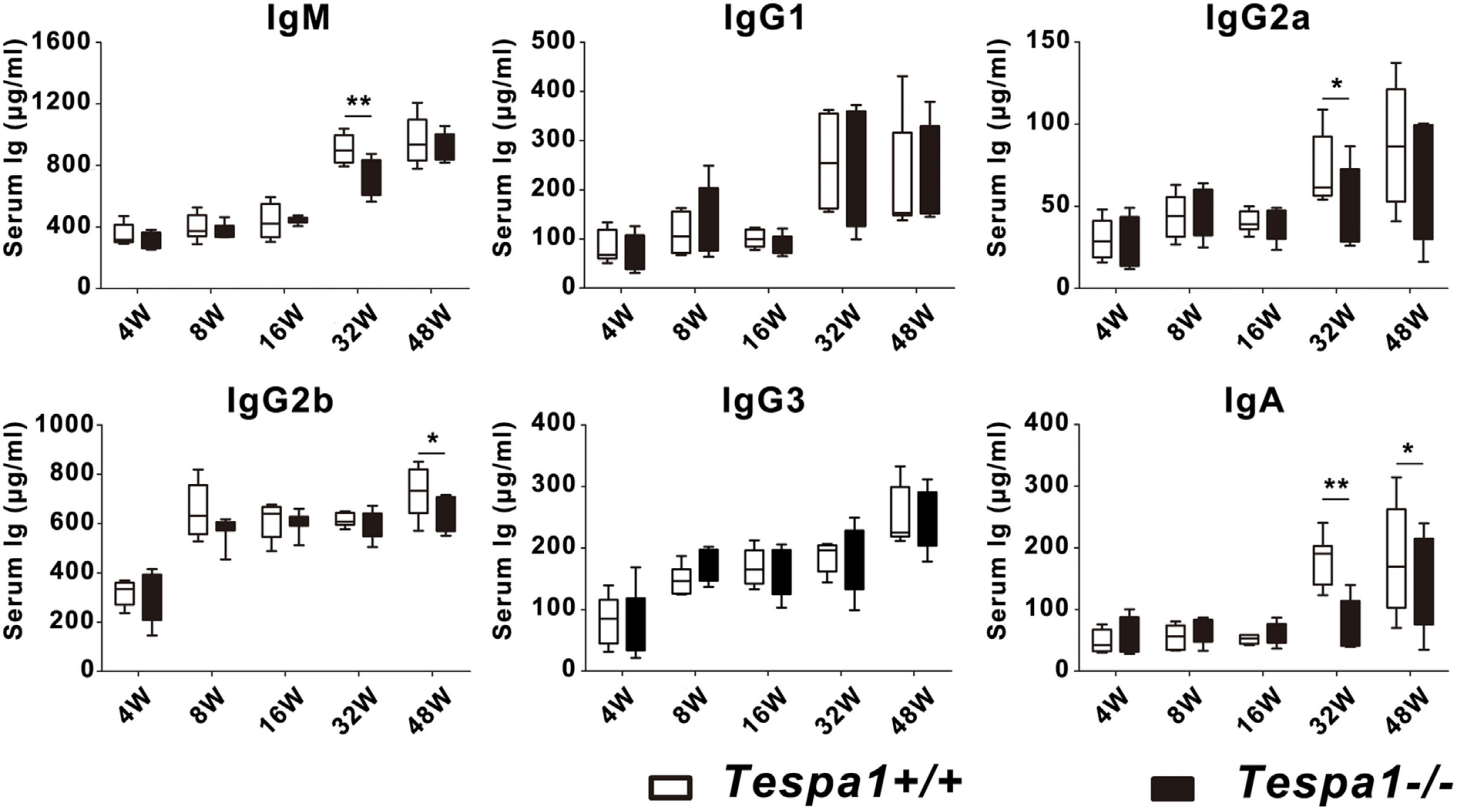
Aged *Tespa1*-deficient mice have lower baseline serum antibody levels. Baseline serum Ig levels in *Tespa1*-deficient and wild-type control mice of the indicated ages, measured by ELISA. Data are shown as the mean ± SEM with eight mice per group and are from one experiment representative of two performed. *p < 0.05, **p < 0.01 (Mann–Whitney test).

To examine the role of *Tespa1* in the acquisition of humoral immunity, we first measured the baseline levels of serum immunoglobulins in aged (32- and 48-week-old) *Tespa1*-deficient mice. We found that the concentrations of most Ig subtypes, including IgM, IgG2a, IgG2b, and IgA, were decreased in aged (32- and 48-week-old) *Tespa1*-deficient mice (Figure 2).

### Decreased TD Humoral Responses in *Tespa1*^B−/−^ Mice

**Figure 3:**
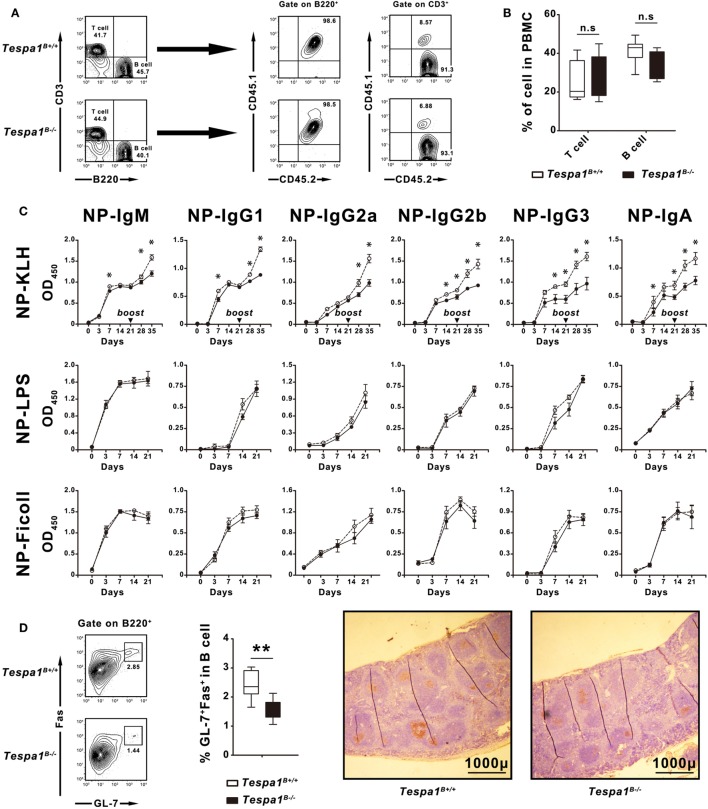
Selective impairment of T cell-dependent responses in *Tespa1*-deficient chimeras. Chimeric mice reconstituted with mixed bone marrow suspensions were generated as described in Section “Materials and Methods.” **(A)** Peripheral blood mononuclear cells (PBMCs) of chimeric mice examined by FACS to identify the origin of the reconstituted lymphocytes. In chimeric mice, 100% of the B cells originate from CD45.1^+^CD45.2^+^ donors, whereas more than 90% of the T cell populations originate from CD45.1^−^CD45.2^+^ μMT mice. **(B)** Percentages of T and B cells in PBMCs of *Tespa1*-deficient and wild-type (WT) chimeras. **(C)** NP-specific serum Ig levels in *Tespa1*-deficient and WT chimeras at different time points after immunization (on day 0) with NP-KLH, NP-LPS, or NP-Ficoll. **(D)** (Left) Splenocytes from *Tespa1*-deficient or WT chimeras immunized with NP-KLH were stained with anti-B220, anti-Fas (CD95), and anti-GL-7 antibodies. Cells were gated on the B220^+^ population. (Right) Immunohistochemical staining with peanut agglutinin (brown) of spleens from control and *Tespa1*-deficient chimeras collected on day 21 after immunization. Scale bar, 1,000 µm. Summarized data shows the mean ± SEM from three separate experiments (*n* = 10 mice per group), *p < 0.05, **p < 0.01.

*Tespa1* plays an important role in various immune cells which are directly or indirectly involved in the development of humoral immunity. To determine whether the reduced concentrations of immunoglobulins seen in *Tespa1*-deficient mice were caused by a functional defect of B cells or other hematopoietic cell types such as T cells, bone marrow chimeric mice were generated. Lethally irradiated C57BL/6 mice were reconstituted with a bone marrow mixture prepared from B cell-deficient μMT mice (CD45.1^−^CD45.2^+^), and either WT C57BL/6 or *Tespa1*-deficient mice (CD45.1^+^CD45.2^+^). These chimeric mice possessed either WT (*Tespa1*^B+/+^) or *Tespa1*-deficient (*Tespa1*^B−/−^) B cells; however, more than 90% of their T cell populations were *Tespa1*-sufficient (Figure 3A). We found no significant alteration in the percentages of T and B cells in the peripheral blood of reconstituted chimeric mice, further confirming that *Tespa1* deficiency does not affect the development of B cells (Figure 3B).

After reconstitution, mice were immunized with the following NP-conjugated antigens: NP-KLH (TD antigen), NP-LPS (TI-1 antigen) and NP-Ficoll (TI-2 antigen), and hapten-specific responses were measured. After immunization with NP-KLH, we detected a reduction of all types of NP-specific Ig responses in *Tespa1*^B−/−^ mice when compared with the *Tespa1*^B+/+^ controls, especially following the secondary immunization. By contrast, no significant differences were seen with TI-antigens (Figure 3C).

Germinal centers are the sites where T–B cell interactions occur during TD B cell responses, and these structures are a hallmark of Th activity (27, 28). To determine whether *Tespa1* deficiency affected GC formation, the spleens of *Tespa1*^B−/−^ and *Tespa1*^B+/+^ mice immunized with NP-KLH were removed at day 21 post-immunization and analyzed by flow cytometry and immunohistochemistry. We found that the percentage of B220^+^Fas^+^GL-7^+^ GC-B cells was significantly higher in immunized *Tespa1*^B+/+^ mice. Staining with PNA also showed enhanced GC formation in *Tespa1*^B+/+^ mice when compared with *Tespa1*^B−/−^ (Figure 3D).

### *Tespa1* Deficiency Impairs Thymus-Dependent B-Cell Activation and Proliferation

**Figure 4:**
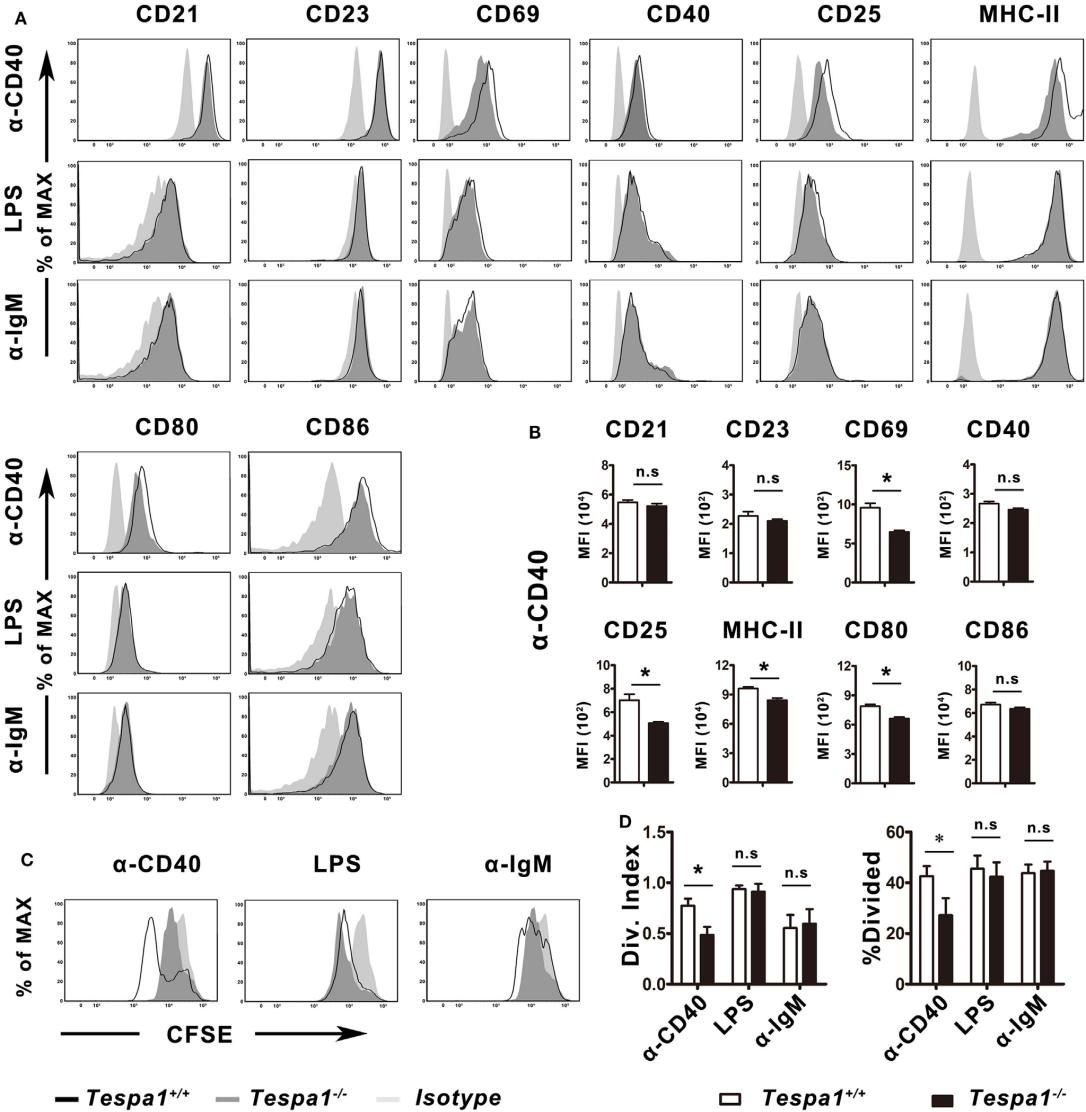
*Tespa1* deficiency selectively decreases CD40-mediated B-cell activation and proliferation. **(A,B)** WT (black) and *Tespa1*-deficient (gray) B cells were stimulated with α-CD40, LPS, or α-IgM F(ab′)_2_. The expression of CD21, CD23, CD44, CD40, CD25, CD69, CD80, CD86, and MHC-II was analyzed by flow cytometry on B220^+^ gated cells after 48 h of stimulation with the indicated molecules. **(C,D)** Carboxyfluorescein succinimidyl ester (CFSE)-stained B cells were stimulated with the indicated antibodies or antigen for 72 h, and proliferation was measured by flow cytometric analysis. Cells were gated on B220^+^. Histogram overlays depict the expression of the indicated markers. Summarized data shows the mean ± SEM from three separate experiments (*n* = 3), *p < 0.05.

To characterize the effect of *Tespa1* on B cell activation at the cellular level, B cells from *Tespa1*^+/+^ and *Tespa1*^−/−^ mice were enriched and stimulated *in vitro* with anti-mouse CD40 antibody (TD response), LPS (TI-1 response) and anti-IgM F(ab′)_2_ (TI-2 response) as described in Section “Materials and Methods.” The surface expression of antigen-presenting molecules (MHC II), costimulatory molecules (CD80 and CD86), and activation markers (CD21, CD23, CD25, CD44, and CD69) was analyzed by flow cytometry (Figure 4). We found that *Tespa1*-deficient B cells expressed lower levels of activation markers (CD25, CD69, CD80, CD86, and MHC-II) following stimulation with anti-CD40 mAb (Figures 4A,B). By contrast, no significant differences were observed after stimulation with anti-IgM F(ab′)_2_ or LPS. In addition, the *in vitro* proliferation, measured with CFSE, was significantly reduced in *Tespa1*-deficient splenic B cells following anti-CD40 mAb stimulation (Figures 4C,D).

### Altered CD40 Signaling Events in *Tespa1*-Deficient B Cells

**Figure 5:**
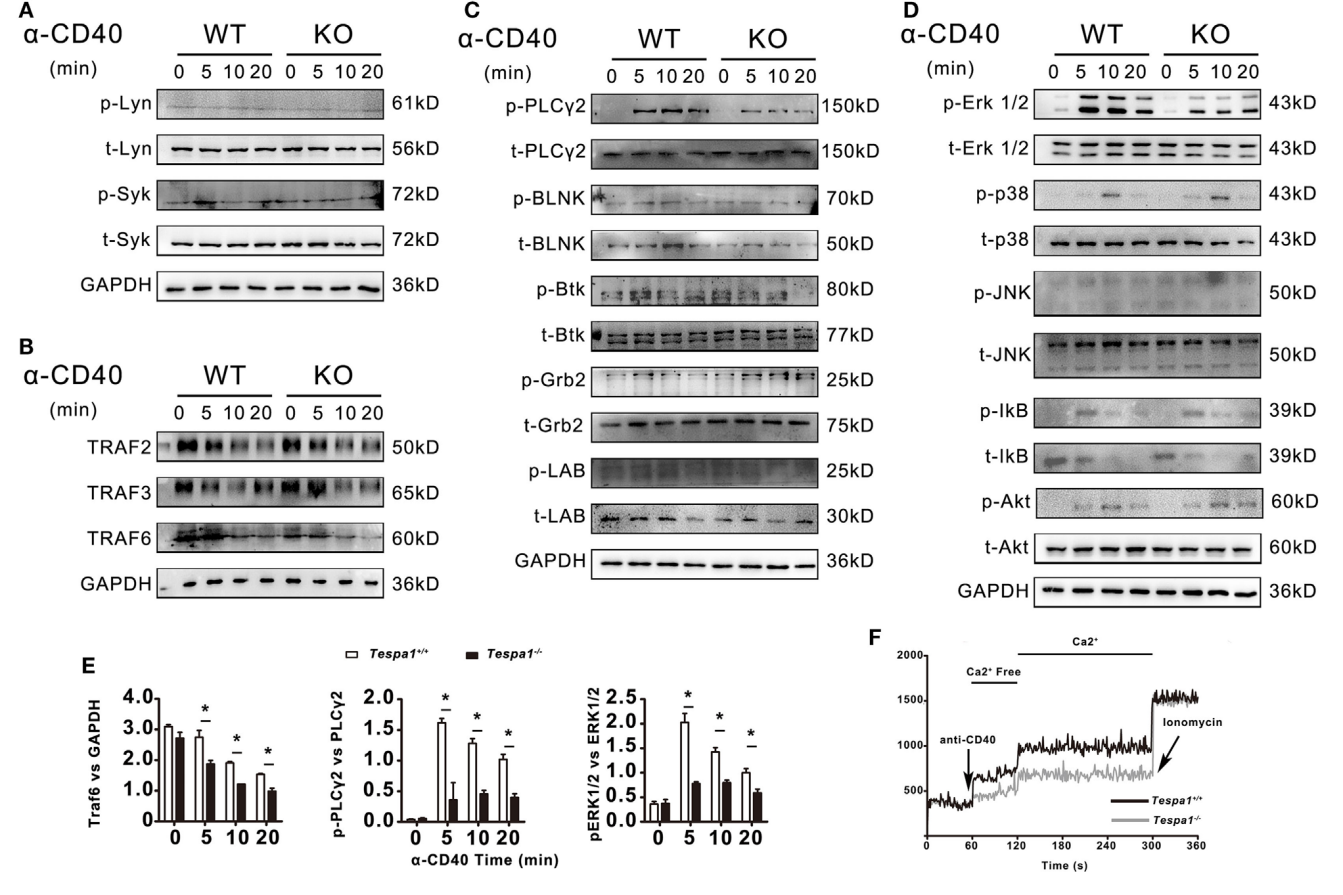
B-cell receptor and CD40-mediated cell signaling in *Tespa1*-deficient and wild-type (WT) B cells. **(A)** Levels of total and phosphorylated Lyn and Syk. **(B)** Levels of TNFR-associated factor (TRAF) 2, TRAF 3, and TRAF 6. **(C)** Levels of total and phosphorylated PLCγ2, BLNK, Btk, Grb2, and LAB. **(D)** Levels of total and phosphorylated Erk, p38, Jnk, Ikb, and Akt were measured by immunoblot. **(E)** TRAF 6 was normalized to GAPDH, Phospho-Plcγ2 and Phospho-ERK1/2 was normalized to total Plcγ2, and total ERK1/2 by densitometry statistical analysis. **(F)** Calcium flux was measured at the indicated times after stimulation. Data are from one experiment representative of three performed.

The above results indicate that *Tespa1* positively regulates thymus-dependent B-cell activation, both *in vitro* and *in vivo*. Next, we examined the activation of intracellular signaling pathways in *Tespa1* wild type (WT) and knockout (KO) B cells. Since we detected differences in B cell activation only for thymus-dependent responses, B cells were stimulated with anti-mouse CD40 for different time periods and analyzed by Western blot. The levels of total (t) and phosphorylated (p) BCR-proximal tyrosine kinases Lyn and Syk were unchanged in B cells derived from *Tespa1* KO mice when compared with WT controls (Figure 5A). In addition, we investigated CD40 signaling mechanisms by examining TRAFs and found that *Tespa1* deficiency impaired the stabilization of TRAF6 but not TRAF2 or TRAF3 following CD40 stimulation (Figures 5B,E). We also examined the levels of phosphorylation of other components of the BCR signalosome, including PLCγ2, BLNK, Btk, Grb2, and LAB, and only found significantly reduced phosphorylation of PLCγ2 in *Tespa1* KO B cells after stimulation (Figures 5C,E). In addition, *Tespa1* deficiency resulted in the attenuated activation of distal signaling mitogen-activated protein kinases ERK (Figures 5D,E), which are widely reported to be critical for B cell activation. These data suggest that the absence of *Tespa1* perturbs a principal signaling axis (CD40/TRAF6/PLCγ2/MAPK) in B cells. A transient increase of intracellular calcium is essential for the activation, proliferation, and differentiation of B cells (29), and the phosphorylation of PLCγ2 could influence calcium influx. As expected, we found that B cells from *Tespa1* WT mice had much higher levels of calcium flux than *Tespa1* KO B cells (Figure 5F). In summary, these data suggest that the positive regulation of B cell activation by *Tespa1* involves modulation of BCR signaling events.

### Decreased Severity of CIA in *Tespa1*-Deficient Chimeras

**Figure 6:**
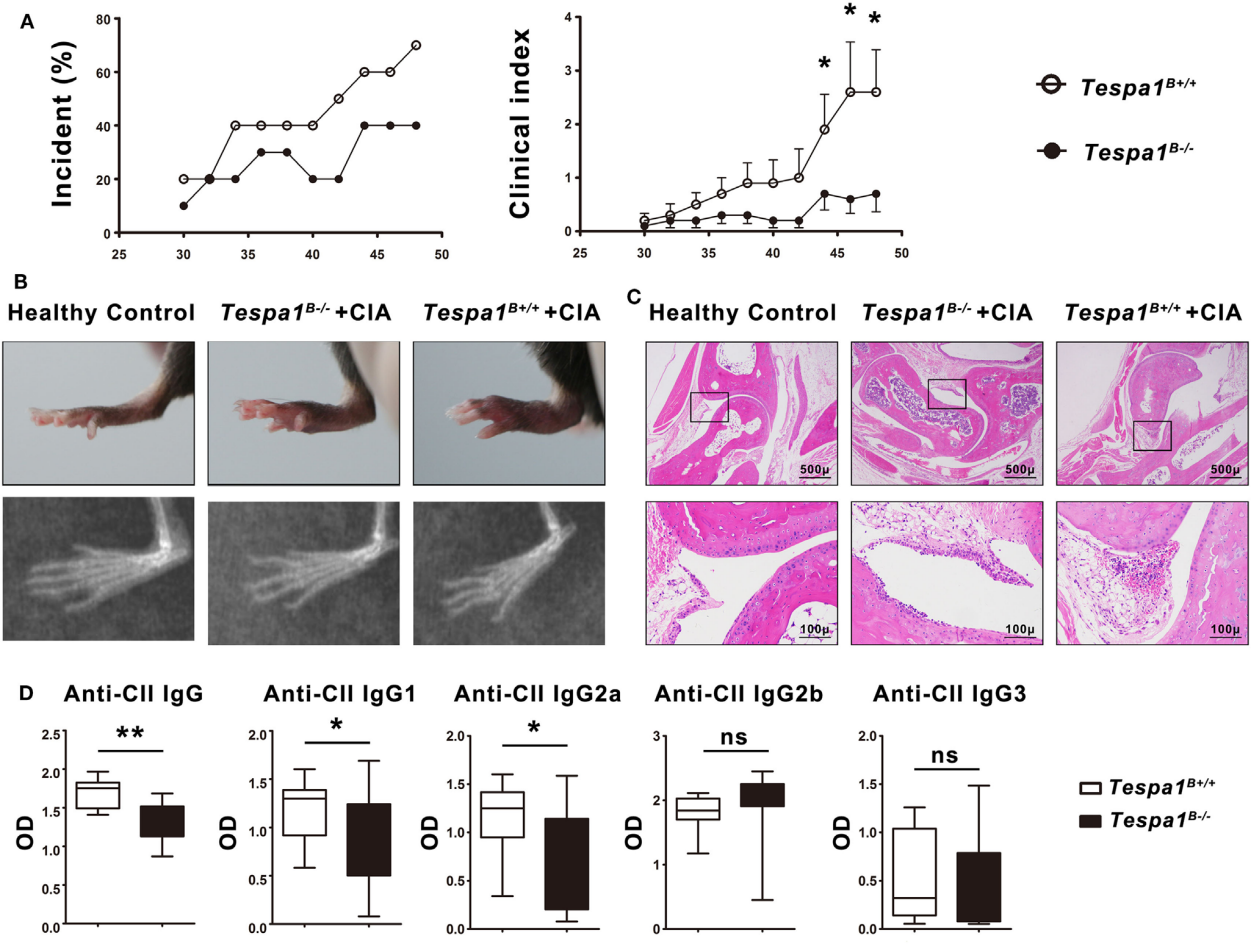
*Tespa1* deficiency significantly attenuates collagen-induced arthritis (CIA) in chimeric mice. **(A)** Incidence and clinical arthritis scores of *Tespa1*-deficient and WT chimeras following experimental induction of CIA. **(B)** Photographs and X-ray images of the hind limbs of WT and *Tespa1*-deficient chimeras 6 weeks after the first CII injection. **(C)** Microscopic sections of the knee joints of *Tespa1*-deficient and WT chimeras stained with H&E. Images are shown at 10× and 50× magnification. **(D)** Anti-collagen II IgG, IgG1, IgG2a, IgG2b, and IgG3 antibody levels in the sera of WT and *Tespa1*-deficient chimeras following CIA induction. Data shown are the mean ± SEM with 10 mice per group and are from one experiment, representative of two performed. *p < 0.05 and **p < 0.01 (Mann–Whitney test).

Because B cells are known to play an essential role during the effector phase of autoimmune arthritis, we examined the progression of CIA, a murine model of human RA, in *Tespa1*^B−/−^ and *Tespa1*^B+/+^ chimeras. The incidence and clinical disease severity index of CIA were significantly reduced in *Tespa1*^B−/−^ chimeric mice when compared with *Tespa1*^B+/+^ chimeric controls (Figure 6A). In the radiological and histologic analysis, we observed more radiological abnormalities and more leukocytic infiltration in the ankle joints of *Tespa1*^B+/+^ than in *Tespa1*^B−/−^ chimeras (Figures 6B,C). Finally, anti-CII IgM, IgG1, IgG2a IgG2b, and IgG3 antibody levels were measured in the chimeric mice by ELISA. Anti-collagen IgG, IgG1, and IgG2a antibody levels were significantly decreased in *Tespa1*^B−/−^ mice, whereas the IgG2b and IgG3 levels did not differ in *Tespa1*^B−/−^ mice (Figure 6D). Taken together, our experiments suggest that *Tespa1* modulates the onset and severity of CIA.

## Discussion

In this study, we found an unexpected role for *Tespa1* in CD40-mediated B cell activation and proliferation. The absence of *Tespa1* did not affect the development of B cells but did impair thymus-dependent B-cell activation, both *in vitro* and *in vivo*.

Optimal B cell activation depends on signals generated by Ag recognition through the BCR, as well as on additional signals provided by cognate interactions with T helper cells, including those triggered by CD40–CD40L interactions (30). The cross talk between CD40 and the BCR modulates the activation of multiple signaling molecules such as PI3K, NF-κB, PLCγ2, and MAPK; and regulates the development, survival, differentiation, and immune responsiveness of B cells (9, 10, 31–33). CD40 is a member of the TNFR family and possesses neither intrinsic kinase activity nor conserved tyrosine residues in its cytoplasmic (CY) tail. Several TRAF members bind to CD40 and appear to function as adaptor proteins (6, 11). TRAF6 is one of the most important TRAFs and contributes to the CD40-mediated activation of B cell. TRAF6 may function as an adapter molecule, an activator of mitogen-activated protein kinases, or act as a repressor of certain signaling circuits (34). In different immune cells, proteasome-dependent TRAF6 degradation has been observed and was reported to impair the downstream signaling pathways leading to cell activation (35–37). However, the molecular mechanisms underlying the stabilization of TRAF6 are not well understood.

Phosphoinositide-specific C phospholipases (PLC) are a critical group of cell-signaling molecular switches that regulate the formation of the second messengers inositol 1,4,5-trisphospate (IP3) and diacylglycerol. There are six families of PLC enzymes, differing in their structural organization and amino acid sequence (38). PLCγ2 is highly expressed in cells of hematopoietic origin and plays a critical role in B cell function (39–41). In some previous reports, PLCγ2 phosphorylation was detected following CD40 ligation, and a complex between TRAF6 and PLCγ2 was also identified in B-cells (9, 10, 42). However, the signaling pathways regulating these activation processes remain poorly understood.

In this study, we found that the absence of *Tespa1* decreased the stabilization of TRAF6, attenuated the phosphorylation of PLCγ2 in anti-CD40-stimulated B cells, and impaired specific distal signaling events, such as the activation of ERK mitogen-activated protein kinases and calcium flux. By contrast, it had no effect on other adaptor proteins, such as BLNK, Btk, and Grb2. These findings imply that *Tespa1* may be involved in the formation of TRAF6-PLCγ2 complexes as reported previously (42), and suggest there is a CD40/TRAF6/*Tespa1*/PLCγ2/MAPK signaling axis in involved in CD40-induced B cell activation.

To elucidate the possible role of *Tespa1* in B cell-related autoimmune diseases, we induced CIA in bone marrow chimeric mice and showed that the development of CIA was clearly attenuated in *Tespa1*-deficient chimeras, suggesting that *Tespa1* may be a potential therapeutic target in human RA.

Our study has limitations and there are still many unanswered questions. We cannot exclude the possibility that *Tespa1* may be involved in the regulation of other B cell signaling pathways since we detected no significant changes in NK-κB, which is the classic protein that is activated downstream of CD40 and TRAF 6. We also did not find the target protein that interacts directly with Tespa1 in B cells.

More importantly, it has been reported that decreased expression of *Tespa1* in peripheral blood mononuclear cell is partially associated with a reduced susceptibility to human RA, but not with disease severity. Since many different types of immune cells are involved in the development of RA (43), and *Tespa1* may also modulate the functions of these immune cells, this may mask its role in B cell thymus-dependent humoral responses.

Taken together, our results suggest that *Tespa1* may be a previously unsuspected missing component of the CD40-proximal signaling machinery in B cells, placing *Tespa1* in a key position to regulate B cell-mediated autoimmune diseases and suggests possible new signaling pathways in B-lymphocytes. Further research along these lines may increase our understanding of the mechanistic relation between human autoimmune disease-associated alleles and B-cell physiology.

## Ethics Statement

This investigation was conducted in accordance with the ethical standards of the Declaration of Helsinki, followed national and international guidelines and was approved by the review board of the School of Medicine, Huzhou University.

## Author Contributions

YY and WH were involved in design, performing and analysis of experiments, and contributed to drafting of the manuscript. XiaoL, XiawL, JQ, and XA were involved in performing of experiments. HH and HS provided resources and were involved in editing the manuscript. LL was involved in the conception of the study. HZ was involved in design of the study.

## Conflict of Interest Statement

The authors declare that the research was conducted in the absence of any commercial or financial relationships that could be construed as a potential conflict of interest.
